# A prospective study on the association between social support perceived by parents of children aged 1–7 years and the use of community youth health care services

**DOI:** 10.3389/fpubh.2022.950752

**Published:** 2022-09-30

**Authors:** Irene N. Fierloos, Dafna A. Windhorst, Yuan Fang, Rienke Bannink, Marlinda Stam, Conny A. A. Slijkerman, Wilma Jansen, Hein Raat

**Affiliations:** ^1^Department of Public Health, Erasmus Medical Center, Rotterdam, Netherlands; ^2^Department of Cognitive Neuroscience, Donders Institute for Brain, Cognition and Behaviour, Radboud University Medical Center, Nijmegen, Netherlands; ^3^TNO Child Health, Leiden, Netherlands; ^4^CJG Rijnmond, Rotterdam, Netherlands; ^5^JONG JGZ, Dordrecht, Netherlands; ^6^Department of Social Development, City of Rotterdam, Rotterdam, Netherlands

**Keywords:** social support, community care, youth healthcare, parenting, empowerment, Health-seeking behavior

## Abstract

**Aim:**

This study examined the association between social support perceived by parents of children aged 1–7 years and the use of additional community youth health care services.

**Methods:**

Data of 749 parents of children aged 1–7 years, gathered in the CIKEO cohort study in the Netherlands, were analyzed. Social support was assessed with the Multidimensional Scale of Perceived Social Support. Data on the use of additional community youth health care services during a period of 1.5 years were obtained from the electronic records of participating youth health care organizations. Multivariable logistic regression models were used to examine the association between perceived social support and the use of additional youth health care services and to explore moderation by the parent's educational level.

**Results:**

The mean age of the responding parents was 33.9 years (SD = 5.1); 93.6% were mothers. Parents who perceived low to moderate levels of social support had 1.72 (95% CI: 1.11, 2.66) times higher odds of using one or more additional youth health care services during the study period compared to parents who perceived high levels of social support at baseline. This association was independent of predisposing factors, but not independent of need factors (*p* > 0.05). Furthermore, the association was moderated by the educational level of the parent (*p* = 0.015). Among parents with a high educational level, low to moderate levels of perceived social support at baseline were associated with 2.93 (95% CI: 1.47, 5.83) times higher odds of using one or more additional youth health care services during the study period independent of predisposing and need factors. Among parents with a low or middle educational level the association between perceived social support and use of additional youth health care services was not significant.

**Conclusion:**

Our findings provide evidence that low to moderate levels of perceived social support are associated with a higher use of additional community youth health care services among parents of children aged 1–7 years, especially among high educated parents. Recommendations for policy and practice are provided.

## Introduction

Many countries offer community youth health care services to monitor and promote children's health and development (1–3). A widely used model on health care utilization, developed by Andersen, Davidson (4) provides insight into the determinants of the use of community youth health care services. According to this model, health care utilization is influenced by individual and contextual factors, each consisting of predisposing, need and enabling factors (4, 5). Although the model emphasizes the importance of contextual factors (4), the influence of the social network on the use of community youth health care services gained little attention in empirical research (6–12).

Gourash (9) proposed that social networks may influence the use of youth health care services in three ways (9). First, by influencing norms and attitudes toward care use, which may reduce or enhance the likelihood that youth health care services are used in case they are needed (9). Second, by providing various types of support including material resources, emotional support, advice, and assistance with child care, which may reduce the need for care (9, 13–16). Third, by the positive outcomes of perceived social support for families' health and wellbeing (17–27). Higher levels of perceived social support have been associated with a higher parenting sense of competence, more positive parenting behavior, better coping mechanisms, and a decreased risk of depression in parents (17–27). By preventing, alleviating and solving problems, social support may influence the use of community youth health care services (9).

Nevertheless, the association between social support and the use of community youth health care services has hardly been examined in empirical studies. Even with regard to other types of youth care and pediatric medical care, empirical studies on the association between social support and care use are scarce and the results of the few studies that were conducted are inconsistent (8, 28–31). Horwitz et al. (31) found no association between social support and the use of pediatric medical care, while Riley et al. (28), found that high levels of perceived social support were correlated with a lower use of pediatric medical care, but not after adjusting for potential confounders. In a study among a low educated “at risk” population (8), parents who perceived high levels of social support were more likely to participate in preventive home visits (8). With regard to the use of mental health care for youth, results of systematic review by Planey et al. (32) and Radez et al. (33) suggest that having a supportive social network may increase the use of mental health care. On the other hand, results of a systematic review by Eijgermans et al. (34) suggest that higher levels of perceived social support may be associated with a lower use of mental health care.

Given the contradictory findings with regard to various types of youth care and the lack of related studies on the use of community youth health care services, more research is need to gain insight into the association between social support and the use of community youth health care services.

Therefore, this study will examine the association between perceived social support by parents of children aged 1–7 years and the use of additional community youth health care services. We hypothesize that parents who perceive low levels of social support use more additional community youth health care services during the study period than parents who perceive high levels of social support (9) (hypothesis 1). In addition, this study explores whether this association is moderated by the parent's educational level. Parents with a higher educational level may have more positive attitudes toward additional community youth healthcare services and may be more proactive in care seeking when informal support is insufficient (31, 35, 36). We hypothesize that the association between social support and the use of additional community youth health care services may be stronger among parents with a high educational level compared to parents with a low educational level (hypothesis 2).

## Materials and methods

### Data collection and study population

This prospective correlational study used data of a cohort study embedded in the Consortium Integration Knowledge promotion Effectiveness Of parenting interventions (CIKEO). The CIKEO project was originally designed to examine the effects of (elements of) parenting support on parent and child outcomes (37).

In the CIKEO project, baseline data were collected between October 2017 and March 2018. Two community youth health care organizations in the areas Rotterdam and Dordrecht used their registries to send invitation letters and questionnaires to parents/caregivers of children aged 1–7 years. In the Netherlands, all children are registered at their local community youth health care organization, regardless of whether they do or do not use youth health care services. All parents who provided written informed consent and returned the questionnaire were enrolled in the study. The parent who generally spends most time with the child was invited to complete the questionnaire. With permission of the parent, additional data were requested from their community youth health care organization's electronic registries. Participation was completely voluntary. A detailed description of the recruitment process has been provided by Windhorst, Fang (37).

**Figure 1:**
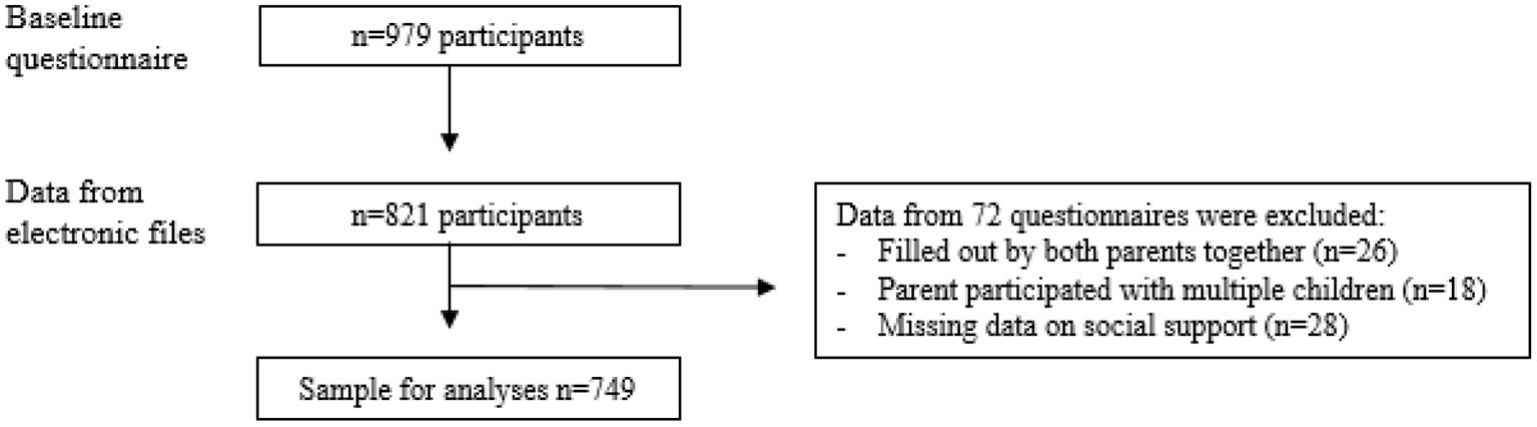
Flowchart of the inclusion process of the CIKEO cohort study and the final sample for analyses (*n* = 749).

In total, 979 parents recruited *via* community youth health care organizations filled out the baseline questionnaire (Figure 1); for 821 parents additional data could be obtained from the youth health care electronic registries. Data from 26 questionnaires completed by two parents together were excluded from the analyses; 18 parents participated in the study with multiple children, and questionnaires filled out for their second child were excluded. Participants with missing information on social support (*n* = 28) were excluded from the analyses. Hence, the population for analyses consisted of 749 participants (Figure 1).

### Measures

#### Additional community youth health care services

In the Netherlands, community youth health care services are freely available to all families (1). From birth onwards, parents regularly receive an invitation to visit a youth health care doctor or nurse with their child; the attendance rate is above 90% (38). During these visits, the health, development, and wellbeing of the child are monitored, and parents receive information and advice about health promotion, child wellbeing and parenting (3, 39). If needed, additional community youth health care visits are scheduled to further examine a specific problem or issue. Parents can also request additional visits or use walk-in consult hours to receive additional advice on any issue related to their child's health, wellbeing or parenting (3).

For parents who gave permission, additional data on the use of youth health care services during a study period of 1.5 years (1st of October 2017 until the 1st of April 2019) were obtained from the electronic registries of the two participating community youth health care organizations. Available data were the total number of visits of the child for whom the questionnaire was completed and the type of visit(s) (regular/additional). Standard age-related visits were categorized as “regular” visits. All other visits, i.e., further examinations, additional appointments for advice and prevention, and walk-in consult hours, were categorized as “additional youth health care services.” For the purpose of this study, we were interested in the use of additional services.

#### Social support

Perceived social support was assessed with the 12-item Multi-dimensional Scale of Perceived Social Support (MSPSS) (40). Previous validation studies showed a high internal reliability among diverse populations, with Cronbach's alpha coefficients ranging between 0.74 and 0.95 (40–44). The MSPSS consists of statements such as: “I get the emotional help and support I need from my family”; “There is a special person in my life who cares about my feelings”; “My friends really try to help me.” Answers were given on a 7-point Likert scale, ranging from 1 = “very strongly disagree” to 7 = “very strongly agree.” The scores were calculated as described in the guidelines (45). Scores ranging from 1 to 2.9 indicated low levels of support, scores ranging from 3 to 5 indicated moderate levels of support, and scores ranging from 5.1 to 7 indicated high levels of support (45). Among our study population, scores below 2.9 were rare (*n* = 3; 0.4%). Therefore, low and moderate MSPSS scores (< 5.1) were categorized as “low to moderate” social support; MSPSS scores ≥5.1 were categorized as “high” social support.

#### Covariates

Potential confounders were categorized as predisposing, need and enabling factors (4). Predisposing factors relate to conditions and characteristics that predispose people to use care, need factors relate to the actual need for care, and enabling factors relate to conditions and resources that facilitate care use (4). The following predisposing factors were included as potential confounders: age (in years), gender of the child (girl/boy), age of the responding parent (in years), gender of the responding parent (female/male), educational level of the responding parent, immigration background of the responding parent, family situation (one-parent family/two-parent family), and the number of children in the household (one/two or more) (4, 28, 29, 46–48). The highest completed educational level of the responding parent was categorized based on the International Standard Classification of Education 2011 (49). Level 0–2 (no education, primary education, lower secondary education) was categorized as “low”; level 3–5 (upper secondary to short-cycle tertiary education) was categorized as “middle”; level 6–8 (bachelor to doctoral) was categorized as “high” (49). When the responding parent or at least one of his/her parents were born outside the Netherlands, this was categorized as having an immigration background (50).

The following need factors were included as potential confounders: the child's general health, the child's emotional and behavioral problems according to their parent, the parent's mental health status, and parenting self-efficacy (30, 47, 51). The general health status of the child was assessed with the first item of the Child Health Questionnaire (52). Scores ranged between 0 (poor health) and 100 (excellent health). The child's emotional and behavioral problems according to their parent were assessed with the Child Behavior Check List (CBCL/1.5–5 year) (53). The CBCL consists of 99 items concerning the child's behavior in the previous 2 months. Each item was scored on a three-point scale with 0 (not true), 1 (somewhat or sometimes true), and 2 (very true or often true). A total problem score was computed by summing up the scores of the 99 items. Scores ranged between 0 (lowest score) and 198 (highest score); higher scores indicate more problems (53). The parent's mental health status was assessed with the Brief Symptom Inventory 18 (BSI-18) (54). The BSI-18 consists of 18-items and three subscales: depression, anxiety, and somatization. One item on thoughts of ending your life was removed from the questionnaire, because it was perceived to be too invasive. Each item was scored on a five point scale ranging from 0 (not at all) to 4 (an awful lot). For the missing item, the respondents' mean was substituted. The total score, indicating general psychological distress, ranged from 0 (lowest score) to 72 (highest score); higher scores indicate more psychological distress. Parenting self-efficacy was measured with the self-efficacy subscale of the 17-item Parenting Sense Of Competence scale (PSOC) (55). The PSOC consists of a self-efficacy and a satisfaction subscale. The 7-item self-efficacy subscale was used to assess parenting self-efficacy on a 6-point Likert scale, ranging from 1 (strongly agree) to 6 (strongly disagree). Scores ranged from 7 (lowest parenting self-efficacy) to 42 (highest self-efficacy) (55).

Enabling factors were not included as potential confounders because community youth health care services in the Netherlands are widely used (acceptability), free of charge (affordability), and usually available nearby (accessibility) (4). However, the frequency, intensity and content of the available community youth health care services may differ by region and therefore a dummy variable indicating the region of the community youth health care center (Dordrecht/Rotterdam) was included as a potential confounder in all regression models (3).

### Ethical considerations

The Medical Ethics Committee of the Erasmus Medical Center in Rotterdam, decided that the rules laid down in the Dutch Medical Research Involving Human Subjects Act (in Dutch: “Wet Medisch-wetenschappelijk Onderzoek met mensen”) did not apply to the research proposal of the CIKEO cohort study (proposal number MEC-2017-432). There were no objections to the execution of this study and results of the study could be submitted to scientific journals (Letter NL/sl/321518; 24/07/2017). The study was registered in the Netherlands Trial Registry as NL7342 (37).

### Data analysis

First, descriptive statistics were used to characterize the sample. Characteristics of parents who used one or more additional youth health care services during a period of 1.5 year were compared to characteristics of parents who did not use any additional youth health care services during this period. For continuous variables, *p*-values were based on independent *T*-tests, and for categorical variables, *p*-values were based on Chi-squared tests.

For the main analysis (hypothesis 1), three logistic regression models were used to examine whether social support at baseline was associated with the use of one or more additional community youth health care services during the study period: a bivariate model (model 1), a model adjusted for predisposing factors (model 2), and a model additionally adjusted for need factors (model 3). Odds Ratios (OR) and 95%-confidence intervals (CIs) were calculated for each factor.

For the additional analysis (hypothesis 2), moderation by the parent's educational level was examined by adding the interaction term (social support^*^educational level) to the fully adjusted logistic regression model. Stratified logistic regression models were conducted in case the interaction effect was significant.

Multiple imputation was used to deal with missing values of the potential confounders. The percentage of missing values ranged between 0.01% (*n* = 1) for gender of the parent and 1.2% (*n* = 9) for emotional and behavioral problems of the child. Five imputed datasets were created for pooled estimates. The regression analyses were repeated in the non-imputed dataset, which gave similar results (data not shown). Data were analyzed in Statistical Package for Social Sciences, version 25 for Windows (IBM SPSS Statistics for Windows, IBM Corp). *p*-Values below 0.05 were considered to be statistically significant.

#### Non-response analysis

The socio-demographic characteristics of participants who were excluded from the sample for analysis due to missing data (*n* = 230) were compared with the socio-demographic characteristics of participants included in the sample for analysis (*n* = 749) using chi-squared tests for categorical variables and *T*-Tests for continuous variables. There were no significant differences (*p* > 0.05).

### Characteristics of the sample

Table 1 presents the characteristics of the study population by the use of one or more additional youth health care services during the study period. The mean age of the responding parents was 33.9 years (SD = 5.1); 93.6% were mothers; the mean age of their child was 3.1 years (SD = 1.8); 15.2% of the parents reported low to moderate levels of perceived social support. Additional youth health care services were more often delivered to younger children (*p* = 0.001), children of parents with an immigration background (*p* < 0.001), children with a poorer general health status (*p* = 0.022), children with more behavioral and emotional problems according to their parent (*p* < 0.001), children of a parent with more mental health problems (*p* < 0.001), and children of a parent who perceived low to moderate levels of social support (*p* = 0.006). On average, participants had 1.7 (SD = 1.1) visits at their community youth health care organization during the study period of 1.5 year and used 0.5 (SD = 1.0) additional services (range 0–10).

**Table 1:** Characteristics of 749 parents of children aged 1–7 years participating in the CIKEO study by the use of additional community youth health care services during the study period of 1.5 year.

	**Additional youth health care services during the study period (1.5 year)**			
	**Total**	**No additional services**	**One or more additional services**	***p*-Values**
	***n* = 749 (100%)**	***n* = 534 (71.3%)**	***n* = 215 (28.7%)**	
	**Mean (SD) *n* (%)**	**Mean (SD) *n* (%)**	**Mean (SD) *n* (%)**	
**Predisposing factors**				
*Age of the child (in years)*	3.1 (SD = 1.8)	3.2 (SD = 1.9)	2.7 (SD = 1.7)	**< 0.001**
*Gender of the child*				0.713
Girl	361 (48.3%)	260 (48.8%)	101 (47.2%)	
Boy	386 (51.7%)	273 (51.3%)	113 (52.8%)	
*Age of the parent (in years)*	33.9 (SD = 5.1)	33.9 (SD = 5.0)	33.8 (SD = 5.4)	0.769
*Gender of the parent*				0.947
Female	700 (93.6%)	499 (93.6%)	201 (93.5%)	
Male	48 (6.4%)	34 (6.4%)	14 (6.5%)	
*Educational level^1^*				0.146
High	411 (55.0%)	299 (56.2%)	112 (52.1%)	
Middle	281 (37.6%)	200 (37.6%)	81 (37.7%)	
Low	55 (7.4%)	33 (6.2%)	22 (10.2%)	
*Immigration background of the parent*				**0.001**
No	658 (88.2%)	483 (90.8%)	175 (81.8%)	
Yes	88 (11.8%)	49 (9.2%)	39 (18.2%)	
*Family situation*				0.422
Two-parent family	709 (94.8%)	503 (94.4%)	206 (95.8%)	
One-parent family	39 (5.2%)	30 (5.6%)	9 (4.2%)	
*Number of children in the household*				0.237
One child	224 (29.9%)	153 (28.7%)	71 (33.0%)	
Two or more children	525 (70.1%)	381 (71.3%)	144 (67.0%)	
**Need factors**				
*General health of the child (0 = worst; 100 = best)*	79.3 (SD = 16.6)	80.2 (SD = 15.7)	76.9 (SD = 18.4)	**0.022**
*Behavioral and emotional problems of the child (0 = lowest; 198 = highest)*	20.0 (SD = 16.7)	18.3 (SD = 14.8)	24.4 (SD = 20.1)	**< 0.001**
*Parenting self-efficacy (7 = lowest; 42 = highest)*	31.9 (SD = 4.2)	32.0 (SD = 4.2)	31.5 (SD = 4.1)	0.165
*Mental health parent BSI (0 = least problems; 72 = most problems)*	5.3 (SD = 6.4)	4.8 (SD = 5.7)	6.7 (SD = 7.7)	**< 0.001**
*Perceived social support*				**0.006**
High	635 (84.8%)	465 (87.1%)	170 (79.1%)	
Low to moderate	114 (15.2%)	69 (12.9%)	45 (20.9%)	

p-Values < 0.05 in bold.

p-Values for continuous variables were based on independent T-tests and p-Values for categorical variables were based on Chi-squared tests.

SD, standard deviation.

Missing values: age of the child n = 4; gender of the child n = 2; age of the parent n = 1; gender of the parent n = 1; education parent n = 2; migration background parent n = 3; family situation n = 1; general health of the child n = 8; behavioral and emotional problems n = 9; parenting sense of competence n = 4; mental health of the parent n = 1.

^1^Educational level ‘High': bachelor, master, doctoral or equivalent; ‘Middle': upper secondary education, post-secondary non-tertiary education, short-cycle tertiary education; ‘Low': no education, primary education, lower secondary education.

## Results

### Social support and the use of additional community youth health care services (hypothesis 1)

Table 2 presents the results of the logistic regression models on the association between perceived social support and the use of one or more additional youth health care services. Model 1 presents the unadjusted association. Model 2, adjusted for predisposing factors, shows that parents who perceived low to moderate levels of social support at baseline had higher odds of using one or more additional youth health care services during the study period (OR: 1.72, 95% CI: 1.11, 2.66) compared to parents who perceived high levels of social support. Model 3 shows that this association was no longer significant after adjusting for need factors (OR: 1.46, 95% CI: 0.92, 2.31).

**Table 2:** Logistic regression models on the association between social support and the use of additional community youth health care services among parents of children aged 1–7 years participating in the CIKEO study (*n* = 749).

	**One or more additional youth health care services during the study period (1.5 year)** ***“yes”** = **215 (28.7%)***		
	**Model 1: Crude model OR (95% CI)**	**Model 2: Adjusted for predisposing factors OR (95% CI)**	**Model 3: Adjusted for predisposing and need factors OR (95% CI)**
*Perceived social support*			
High	ref.	ref.	ref.
Low to moderate	**1.78 (1.18, 2.69)**	**1.72 (1.11, 2.66)**	1.46 (0.92, 2.31)
**Predisposing factors**			
*Age of the child (in years)*		**0.84 (0.76, 0.94)**	**0.81 (0.73, 0.91)**
*Gender of the child*			
Girl		ref.	ref.
Boy		1.03 (0.74, 1.43)	0.99 (0.71, 1.38)
*Age of the parent (in years)*		1.02 (0.98, 1.06)	1.03 (0.99, 1.07)
*Gender of the parent*			
Female		ref.	ref.
Male		0.87 (0.43, 1.74)	0.83 (0.40, 1.71)
*Educational level ^1^*			
High		ref.	ref.
Middle		1.16 (0.81, 1.66)	1.08 (0.75, 1.56)
Low		1.76 (0.95, 3.28)	1.63 (0.86, 3.07)
*Immigration background parent*			
No		ref.	ref.
Yes		**2.08 (1.30, 3.35)**	**1.90 (1.16, 3.10)**
*Family situation*			
Two-parent household		ref.	ref.
One-parent household		0.62 (0.28, 1.40)	0.62 (0.27, 1.41)
*Number of children in the household*			
Two or more children		ref.	ref.
One child		1.12 (0.77, 1.62)	1.06 (0.71, 1.56)
**Need factors**			
*General health of the child (better)*			0.99 (0.98, 1.00)
*Behavioral or emotional problems child (more)*			**1.02 (1.00, 1.03)**
*Parenting sense of competence (higher)*			1.01 (0.96, 1.05)
*Mental health of the parent (worse)*			1.02 (0.99, 1.04)

Table is based on the imputed dataset. p-Values < 0.05 in bold. ORs and 95% confidence intervals were derived from the multivariable logistic regression models.

OR, odds ratio; CI, confidence interval; ref., reference group.

All models were adjusted for the region of the community youth health care center.

#### Moderation by educational level (hypothesis 2)

Results of the interaction analysis (Supplementary Table S1) showed that the association between social support and the use of one or more additional youth health care services was moderated by the educational level of the parent (*p* = 0.015).

Table 3 presents perceived social support and the use of additional youth health care services by parents with different educational levels. For the stratified regression analysis, the groups of parents with a middle and low educational level were combined due to the relatively low number of participants with a low educational level (*n* = 55). Table 4 presents the stratified logistic regression models on the association between perceived social support and the use of one or more additional youth health care services. Among parents with a high educational level (*n* = 412), low to moderate levels of perceived social support at baseline were associated with 2.93 times higher odds of using one or more additional community youth health care services during the study period (95% CI: 1.47, 5.83). Among parents with a low/ middle educational level (n = 337), perceived social support was not associated with the use of one or more additional youth health care services during the study period (OR: 0.84, 95% CI: 0.44, 1.62).

**Table 3:** The use of additional youth health care services during the study period among parents of children aged 1–7 years participating in the CIKEO study (*n* = 749) by educational level and perceived social support.

	**Parents with a high educational level (*****n*** = **411)**^**1**^		**Parents with a middle educational level (*****n*** = **281)**^**2**^		**Parents with a low educational level (*****n*** = **55)**^**3**^	
	**No additional services *n* (%)**	**One or more additional services *n* (%)**	**No additional services *n* (%)**	**One or more additional services *n* (%)**	**No additional services *n* (%)**	**One or more additional services *n* (%)**
Perceived social support “high”	272 (75.8%)***	87 (24.2%)***	169 (71.6%)	67 (28.4%)	22 (57.9%)	16 (42.1%)
Perceived social support “low to moderate”	27 (51.9%)***	25 (48.1%)***	31 (68.9%)	14 (31.1%)	11 (64.7%)	6 (35.3%)

Table is based on the non-imputed dataset. Missing values education parent n = 2. Differences between groups were evaluated with Chi-squared tests.

***p < 0.001.

^1^Among parents with a high educational level n = 361 (87.6%) perceived high levels of social support and n = 52 (12.6%) perceived low to moderate levels of social support.

^2^Among parents with a middle educational level n = 236 (84.0%) perceived high levels of social support and n = 45 (16.0%) perceived low to moderate levels of social support.

^3^Among parents with a low educational level n = 38 (69.1%) perceived high levels of social support and n = 17 (30.9%) perceived low to moderate levels of social support.

**Table 4:** Stratified logistic regression models on the association between social support and the use of additional community youth health care services during the study period (1.5 year) among parents of children aged 1–7 years participating in the CIKEO study (*n* = 749); stratified by educational level.

	**Parents with a high educational level (*****n*** = **412)**			**Parents with a low/ middle educational level (*****n*** = **337)**		
	***One or more additional youth health care services during the study period (“yes” n** = **112)***			***One or more additional youth health care services during the study period (“yes” n** = **103)***		
	**Model 1: Crude model OR (95% CI)**	**Model 2: Adjusted for predisposing factors OR (95% CI)**	**Model 3: Adjusted for predisposing and need factors OR (95% CI)**	**Model 1: Crude model OR (95% CI)**	**Model 2: Adjusted for predisposing factors OR (95% CI)**	**Model 3: Adjusted for predisposing and need factors OR (95% CI)**
**Perceived social support**						
High	ref.	ref.	ref.	ref.	ref.	ref.
Low to moderate	**2.91 (1.60, 5.27)**	**3.33 (1.75, 6.36)**	**2.93 (1.47, 5.83)**	1.07 (0.59, 1.95)	1.02 (0.55, 1.89)	0.84 (0.44, 1.62)
**Predisposing factors**						
*Age of the child (in years)*		**0.85 (0.73, 0.98)**	**0.83 (0.71, 0.97)**		**0.84 (0.72, 0.98)**	**0.79 (0.67, 0.93)**
**Gender of the child**						
Girl		ref.	ref.		ref.	ref.
Boy		0.83 (0.53, 1.32)	0.79 (0.49, 1.28)		1.33 (0.82, 2.14)	1.28 (0.78, 2.08)
*Age of the parent (in years)*		1.02 (0.97, 1.08)	1.03 (0.97, 1.09)		1.01 (0.96, 1.07)	1.03 (0.97, 1.08)
**Gender of the parent**						
Female		ref.	ref.		ref.	ref.
Male		0.84 (0.35, 2.03)	0.88 (0.35, 2.22)		0.79 (0.23, 2.75)	0.66 (0.18, 2.49)
**Immigration background parent**						
No		ref.	ref.		ref.	ref.
Yes		**2.48 (1.32, 4.66)**	**2.24 (1.16, 4.33)**		1.83 (0.86, 3.88)	1.75 (0.79, 3.85)
**Family situation**						
Two-parent household		ref.	ref.		ref.	ref.
One-parent household		0.21 (0.03, 1.77)	0.19 (0.02, 1.76)		0.88 (0.35, 2.17)	0.87 (0.35, 2.20)
**Number of children in the household**						
Two or more		ref.	ref.		ref.	ref.
One		**1.89 (1.11, 3.20)**	**1.89 (1.09, 3.29)**		0.64 (0.37, 1.12)	0.60 (0.33, 1.07)
**Need factors**						
*General health of the child (better)*			0.99 (0.98, 1.00)			0.99 (0.98, 1.01)
*Behavioral or emotional problems of the child (more)*			**1.03 (1.01, 1.05)**			1.01 (1.00, 1.03)
*Parenting self-efficacy(higher)*			1.03 (0.97, 1.10)			0.99 (0.93, 1.05)
*Mental health of the parent (worse)*			1.02 (0.98, 1.07)			1.01 (0.98, 1.05)

p-Values < 0.05 in bold. ORs and 95% confidence intervals were derived from the multivariable logistic regression models.

OR, odds ratio; CI, confidence interval; ref., reference group. Table is based on the imputed dataset. All models were adjusted for the region of the community youth health care region.

## Discussion

Low to moderate levels of perceived social support among parents of children aged 1–7 years were associated with higher odds of using one or more additional youth health care services during the study period independent of predisposing factors, but not independent of need factors. The association was moderated by the educational level of the parent. Among parents with a high educational level, low to moderate levels of social support were associated with higher odds of using one or more additional youth health care services, independent of predisposing and need factors. Among parents with a low or middle educational level, social support was not associated with the use of additional youth health care services.

As described in the introduction, the association between social support and the use of community youth health care services has hardly been examined in empirical research. With regard to other types of youth care and pediatric medical care, previous studies found little support for an association between social support and care use (28–31). However, the comparability of these studies may be limited as these studies examined different types of care and used different measures to assess social support (28–31).

We found support for our hypothesis that the association between social support and the use of additional community youth health care services was moderated by the parent's educational level. Among parents with a high educational level, low social support was associated with higher odds of the use of additional community youth health care services during the study period, while there was no association among parents with a low or a middle educational level. Parents with higher educational levels may have more positive attitudes toward (additional) community youth health care services and may therefore be more likely to use these services in case these are needed (31, 35, 36). They may also be more proactive in asking for additional community youth health care services when informal social support is insufficient (31, 35, 36). According to a qualitative study by Turnbull, Pope (56), factors related to a parent's socioeconomic position, like owning a car and having flexible work hours may facilitate the use of health care, while relying on public transportation and inflexible work hours may be a barrier to the use of healthcare (56). The threshold to use additional youth health care services in case they are needed may be lower among parents with a high socioeconomic position (of which the parent's educational level is an important indicator). We recommend to take a broad range of factors into account in future studies. More quantitative research is needed to confirm the moderation effect between social support and educational level. Qualitative research is needed to gain more in-depth insight into factors that possibly explain this moderation effect.

In additional interaction analyses (Supplementary Table 1), we explored possible moderation by other factors, namely the age group of the child (1–3 and 4–7 years) and the immigration background of the parent (57). The analyses showed that these factors were not moderating the association between social support and the use of additional youth health care services (*p*-values of the interaction terms were 0.441 and 0.665, respectively). However, the number of participants with an immigration background was rather low (*n* = 88), and therefore we recommend to examine this moderation effect in future studies. Cultural beliefs and practices, language barriers and familiarity with the health care system might influence the use of community youth health care services in various ways (57, 58).

### Methodological considerations

Strengths of this study include the use of prospective data from the electronic registries of community youth health care organizations and the relatively large sample size. There are also limitations. First, a comparison of the participants' socio-demographic characteristics with national open data (62) showed that parents with a low educational level, parents with a migration background and one-parent families were relatively underrepresented in the sample. We have no rationale to expect that the directions of the associations have been affected by this underrepresentation, however it may have resulted in an over- or underestimation of the strength of the associations. Therefore, we advise to include a more diverse group of parents in future studies. Second, there were no data available on the reasons for the use of additional youth health care services (e.g., physical development or parenting issues) and we did not know whether the additional support was requested by the parent or initiated by the professional. These factors may be included in future studies, together with other potentially relevant factors such as characteristics of the social network, socioeconomic factors and norms and attitudes toward care use (9, 31, 35). Third, this study was not designed to examine causality between social support and the use of additional youth health care services. It may be possible that the use of youth health care services is influencing a parent's level of perceived social support. To reduce this possibility, we used a measure that specifically focused on perceived social support provided by family, a special person and friends, and not on external social support (40). A methodological consideration is that all regression models were adjusted for the region of the community youth health care organization. The region of the community youth health care organization was not significant in any of the regression models and adjustment for this potential confounder did not influence our results. As a final methodological consideration we want to point out that our full multivariable regression models were adjusted for need factors, which may have caused overadjustment. As mentioned in the introduction, need factors are also influenced by social support and may therefore (partly) mediate the association between social support and the use of additional youth health care services (63–67).

### Implications for practice and policy

First of all, our results indicate that parents with a high educational level may be more likely to find their way to additional services in case they perceive low to moderate levels of social support. To ensure that all parents receive appropriate care we advise youth health care professionals to pay attention to all parents who perceive low to moderate levels of social support, and in particular to parents with low/ middle educational levels as our results indicate they may less often find their way to additional youth healthcare services.

In addition, our results have implications for social policy. In several countries, recent policy reforms aim to build stronger informal social networks in order to empower parents and to reduce the demand for youth and family care (16, 59–61). This study provides some empirical support for the association between social support and the use of additional youth health care services. Intervention strategies aiming to strengthen social support may be used to improve families' health and wellbeing and to empower parents to solve parenting issues within their social networks (13–27).

## Conclusion

Our findings support the hypotheses that low levels of perceived social support by parents of children aged 1–7 years are associated with a higher use of community youth health care services, especially among high educated parents. This underlines the relevance of examining the broader social context in which care use takes place. More empirical research is needed to gain a better understanding of the association between social support and the use of community youth health care services and possible moderation effects. To ensure that all parents receive appropriate care, youth health care professionals are advised to pay attention to all parents who perceive low to moderate levels of social support, and in particular to parents with lower educational levels because they may less often find their way to additional youth health care services. We advise to be aware of the relevance of perceived social support for improving families' health and wellbeing and empowering them to solve parenting issues within their social networks.

## Data availability statement

The datasets presented in this article are not readily available because of ethical restrictions. Data that support the findings of this study are available from the corresponding author upon a reasonable request. Requests to access the datasets should be directed at: h.raat@erasmusmc.nl.

## Ethics statement

The studies involving human participants were reviewed and approved by the Medical Ethics Committee of the Erasmus Medical Center in Rotterdam, decided that the rules laid down in the Dutch Medical Research Involving Human Subjects Act (in Dutch: Wet Medisch-wetenschappelijk Onderzoek met mensen) did not apply to the research proposal of the CIKEO cohort study (proposal number MEC-2017- 432). There were no objections to the execution of this study and results of the study could be submitted to scientific journals (Letter NL/sl/321518; 24/07/2017). The study was registered in the Netherlands Trial Registry as NL7342. The patients/participants provided their written informed consent to participate in this study.

## Author contributions

IF: data collection, conceptualization, analysis, and writing—original draft. DW: data collection, supervision, interpretation of the data, and critical review. YF: data collection and critical review. HR: data collection, supervision, interpretation of the data, and critical review. RB, MS, CS, and WJ: data collection and critical review. All authors approved the final version.

## Funding

The CIKEO study was funded by a research grant (project number: 729300015) from ZonMw, The Netherlands Organization for Health Research and Development. The funders had no role in the design and conduct of the study; collection, management, analyses, or interpretation of the data; preparation, review, or approval of the manuscript; and decision to submit the manuscript for publication.

## Conflict of interest

The authors declare that the research was conducted in the absence of any commercial or financial relationships that could be construed as a potential conflict of interest.

## Publisher's note

All claims expressed in this article are solely those of the authors and do not necessarily represent those of their affiliated organizations, or those of the publisher, the editors and the reviewers. Any product that may be evaluated in this article, or claim that may be made by its manufacturer, is not guaranteed or endorsed by the publisher.
