# Supplementary material for: A prospective study on the association between social support perceived by parents of children aged 1–7 years and the use of community youth health care services
Source: Front Public Health. 2022 Sep 30;10:950752. doi: 10.3389/fpubh.2022.950752 (PMC9561893; doi:10.3389/fpubh.2022.950752)
Supplement: Supplementary file 1 [file Table_1.pdf]

Supplementary Table S1. Results of the analyses of interaction effects between social support and the use of one or more additional community youth health care services **during the study period (1.5 year)** among parents of children aged 1-7 years participating in the CIKEO study (n=749).

**Interaction term**	**P-value of the Beta in the fully adjusted multivariable logistic regression model on the association between social support and the use of one or more additional youth health care services).**
Low to moderate social support*educational level	**.015**
Low to moderate social support*immigration background	.665
Low to moderate social support*age group of the child (1-3 years vs. 4-7 years)	.441

Table is based on the imputed dataset. P-values were derived by separately adding the interaction terms to the fully adjusted multivariable logistic regression model on the association between social support at baseline and the use of one or more additional youth health care services during the study period (Table 2; model 3).
